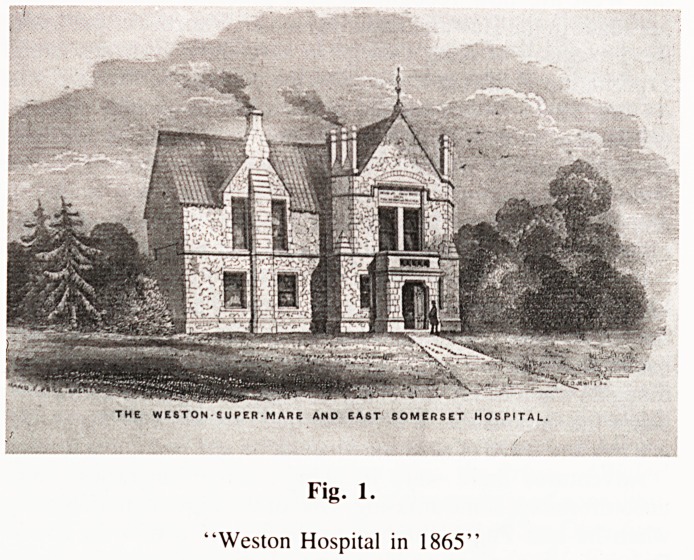# History of the Medical Services in Weston-Super-Mare

**Published:** 1991-12

**Authors:** Hugh Roberts


### History of the Medical Services in Weston-super-Mare

**Hugh Roberts MB, ChB, FRCS**

There is evidence of human settlement in Weston-super-Mare through the old and new stone ages into Roman times. The name Weston does not feature in the Domesday Book, but Weston-super-Mare first appears in the Bath and Wells registry in 1348. The oldest buildings in the area are at Woodspring Priory, founded in 1210. Amongst these buildings is an Infirmary, built in the 15th century. Evidence suggests that such buildings were used for looking after monks, treatment included regular bleeding but there is no evidence of treatment of outsiders.

Woodspring Priory fell into disuse in 1537 but after this there are entries in the local Parish records referring to contributions towards "the Hospital for wounded soldiers at Woodspring". The last recorded payment was in 1734.

The first known doctor practising in Weston was Dr. Richard Tuckey, resident between 1680 and 1740. In 1716 a Dr. Wallan was paid 2/- for "curing Hopkin's child". There are further references to practising doctors and surgeons in the 18th century. Although nothing is known of the practice of these doctors in Weston during the 18th century, there are detailed records of the practice of doctors in Wells at this time. These records suggest that certainly during the latter part of the 18th century the doctors made a good living, Dr. Benjamin Pulsford is credited with earning £400 in 1857.

A Poor House was built in Weston In 1821 and enlarged in 1831. Dr. Samuel Parsley, was the Surgeon Apothecary at Banwell and was on the staff of the Poor House. Between 1821 and 1831 he received over £150 in payment for his services from the Weston Overseers. This included six payments, each for the treatment of a broken leg.

At the beginning of the 19th century, the population of Weston was less than 150 whereas by the mid 1850's it had reached almost 8,000. The expansion in the population was in large part due to the holiday influx, sea bathing had become popular during the latter part of the 18th century. During the 1830's-1850's a number of hotels and large houses were built in Weston together with a large number of houses for the poorer members of society. The railway arrived in Weston in 1841, adding to the prosperity.

The first medical establishment was started by Dr. Edward Long Fox on Knighstone Island, where he treated psychiatric patients. Dr. Long Fox eventually sold this establishment in 1860, although he is commemorated in Weston in the Edward Long Fox Psychiatric Hospital.

A Dispensary was first opened in Weston in 1854 but was closed by the end of the year. A further Dispensary was opened in 1857 and this appeared to thrive. However, there was no Hospital accommodation for patients who were either ill or injured; those that were fit enough being transferred to Bristol.

On the 5th October 1863, a public Tea and Soiree took place in the Town Hall for the purpose of enabling all classes to contribute towards a Casualty Ward Fund, and rightheartedly it was responded to. The Venerable Archdeacon Brown in his speech enumerated the advantages of having a Hospital in Weston. The patient would not be subjected to the agony of a long journey in a railway carriage or a cart, the pure air of the country was much more favourable for the cure of those who had met with injuries and the great excitement connected with a large Hospital, which in some cases could have a very bad effect on the patient, would be avoided if the patient was treated in a local establishment.

Money was obtained and the Hospital built in Great Alfred Street at a cost of £1,295.3s.2d. and opened in 1866. The staff at the start consisted of a Consultant Surgeon, Mr. Richard Alford, an Honorary Physician Dr. F. Gourlay, the Honorary Surgeons Mr. Edward Martin and Mr. C. B. Hitchens, the House Surgeon Mr. E. Scott-Jones, the Dentist Mr. E. T. Drew

**Fig. 1.**

"Weston Hospital in 1865"

and the Matron, Mrs. Rees. During the first year there were seven beds in the Hospital and there were 21 in-patients, including one man who had fallen 60 feet from the New Pier, which was under construction. Had this man had to be transferred to Bristol, he would have had little chance of survival! In 1866-1868 a Medical Wing was added, bringing up the number of beds to 16 and Contagious Wards were constructed in 1870 bringing the beds up to 20. Further expansion occurred in 1877 and by 1879 there were 31 beds. The last extension was in 1887, the new accommodation providing a large comfortable waiting room for the patients, two comfortable rooms for the surgeons whilst above these rooms was a well constructed ward for medical cases.

The Chairman, in his remarks at an Annual General Meeting, records his pride at the cleanliness of the Hospital — "our Hospital is indeed a blessed institution and is kept so clean that without any fastidiousness one could almost eat off the very floors". However, the reports of the visitors and the complaints of the medical staff did not always support these sentiments. It appears that there were considerable problems with smoking chimneys, smelling drains and the buildings were often cold. Amongst his other duties the House Surgeon was expected to visit patients in their homes. In 1877 he made 4,400 such visits. This was clearly too much for him and in 1886 a further Provident Dispensary was established, this remaining open until 1902, the closure being due to financial difficulties.

During the latter part of the 19th century, financial troubles caused the closure of two of the wards. During the early part of the 20th century there were further small extensions and an X-ray room was provided in 1912.

During the First World War ten beds were set aside for military cases. In 1926 a foundation stone was laid for the great new extension which was opened in 1928. This is another story.

## Figures and Tables

**Fig. 1. f1:**